# Technical pre‐analytical effects on the clinical biochemistry of Atlantic salmon (*Salmo salar* L.)

**DOI:** 10.1111/jfd.12476

**Published:** 2016-05-04

**Authors:** M Braceland, K Houston, A Ashby, C Matthews, H Haining, H Rodger, P D Eckersall

**Affiliations:** ^1^ The Centre For Aquaculture TechnologiesSouris, Prince Edward IslandCanada; ^2^ School of Veterinary MedicineCollege of MedicalVeterinary and Life SciencesUniversity of GlasgowGlasgowUK; ^3^ Fish Vet GroupInvernessUK; ^4^ Institute of BiodiversityAnimal Health and Comparative MedicineCollege of MedicalVeterinary and Life SciencesUniversity of GlasgowGlasgowUK; ^5^ Present address: Center for Aquaculture Technologies20 Hope StreetSourisMBCanada

**Keywords:** clinical biochemistry, diagnostics, pre‐analytical, reference range, salmon, Biochemistry, Non‐destructive, Health monitoring, Pre‐analytical effects

## Abstract

Clinical biochemistry has long been utilized in human and veterinary medicine as a vital diagnostic tool, but despite occasional studies showing its usefulness in monitoring health status in Atlantic salmon (*Salmo salar* L.), it has not yet been widely utilized within the aquaculture industry. This is due, in part, to a lack of an agreed protocol for collection and processing of blood prior to analysis. Moreover, while the analytical phase of clinical biochemistry is well controlled, there is a growing understanding that technical pre‐analytical variables can influence analyte concentrations or activities. In addition, post‐analytical interpretation of treatment effects is variable in the literature, thus making the true effect of sample treatment hard to evaluate. Therefore, a number of pre‐analytical treatments have been investigated to examine their effect on analyte concentrations and activities. In addition, reference ranges for salmon plasma biochemical analytes have been established to inform veterinary practitioners and the aquaculture industry of the importance of clinical biochemistry in health and disease monitoring. Furthermore, a standardized protocol for blood collection has been proposed.

## Introduction

Atlantic salmon are susceptible to a number of diseases of known and unknown aetiology. These diseases include various fungal, viral, bacterial and parasitic pathogens which significantly reduce productivity, sustainability and profitability of the industry. While mortality and morbidity resulting from infection and disease is highly variable and dependent on a number of factors (Yousaf *et al*. 2013), it represents a sustainability problem for the industry (Kibenge *et al*. 2012). However, it is well established that disease impacts can be reduced significantly through regular health monitoring of stocks (Raja & Jithendran 2015), with implementation of treatments, and disease management strategies.

The tools for diagnosing infection and disease in finfish aquaculture have grown significantly in recent years (Adams & Thompson 2012). The use of established diagnostic tools such as aetiological agent isolation, PCR and serology has dramatically increased. This is in part because the salmon aquaculture industry is moving away from reliance on behavioural signs of disease to a more proactive approach. However, it is widely established that aetiological agents can be present for a period of time before clinical signs are observed (Kristoffersen *et al*. 2009; Jansen *et al*. 2010). In terms of disease management strategies, this is problematic as the onset of a disease outbreak cannot be determined using current widely used non‐destructive tests. Therefore, there is a heavy reliance on histopathology to identify the clinical manifestations of disease in salmon production. However, histopathology is a laborious and time‐consuming process, which takes a high level of skill for proper assessment. In addition, direct costs are increased by the effect of fish being removed from the population for tissue sampling and therefore not going to harvest. In addition, due to the sequential pathology of many diseases, such as pancreas disease (PD), all disease indicators may not be visible at the same time point (McLoughlin *et al*. 2002). Repeat sampling may be required over a period of time, adding to cost, and there is a distinct fish welfare issue associated with such reliance on destructive sampling. Thus, there is significant demand within the industry for non‐destructive means of health assessment capable of diagnosing clinical disease while also allowing for investigation of higher sample numbers.

Clinical biochemistry is the analysis of concentrations of numerous proteins, metabolites, enzymes and electrolytes in bodily fluids, most commonly blood‐derived serum or plasma, for non‐destructive diagnosis and monitoring of disease. While such an approach is well established in humans and in domestic, laboratory and livestock species (Braun *et al*. 2015), very little work has investigated its usefulness in salmon aquaculture. Despite this, studies in salmon have found creatine kinase (CK) to increase in serum activity during PD (Ferguson, Rice & Lynas 1986; Rodger *et al*. 1991), and lactate dehydrogenase (LDH) and CK to significantly increase due to inflammation and necrosis during heart and skeletal muscle inflammation (Yousaf & Powell 2012). However, the impact of these findings has been restricted by limited understanding of the decision point when results of these enzyme tests become clinically significant. The CK values in these studies were highly variable, and reference ranges, which are imperative for effective identification of disease problems, have not been established. This variability may be due in part to numerous pre‐analytical biological factors, which in human studies have been shown to account for the majority of laboratory errors influencing clinical decisions (Braun *et al*. 2015). In addition, there have, to date, been no attempts to establish standardized sampling protocols for collection of serum or plasma from captive salmon. Furthermore, the effect of technical pre‐analytical factors such as sampling technique and specimen management has not been studied in salmon. Such work is pivotal to the implementation of clinical biochemistry to the salmon aquaculture industry in order to prevent incorrect classification of health status, to provide reliable estimates of disease prevalence and crucially to ensure that laboratory results are reliable.

Therefore, this study aimed to investigate technical pre‐analytical effects on clinical biochemical analysis to assess how treatment of blood samples from salmon can influence measurement of their biochemistry profiles. In total, 22 analytes were assayed in multiple samples in distinct investigations covering the effects of sample type (serum or plasma), the time before separation from blood cells, storage conditions and freeze–thaw cycles. In addition, the study also aimed to establish the degree of individual variation within populations and if pre‐analytical treatments could cause false diagnosis of disease. Moreover, a standardized methodology of sampling and specimen care is proposed that is in line with established protocols for use in other livestock production sectors but not, so far, in aquaculture (Humann‐Ziehank & Ganter 2012).

## Material and methods

### General methods

This study focussed on establishing technical pre‐analytical effects on clinical biochemistry parameters. Each investigation has different sample handling procedures prior to clinical biochemical analysis. Fish were sampled during a routine health monitoring programme (Fish Vet Group, Inverness, UK) from two separate production sites in the west of Scotland with no reported disease problems. Residual blood was used for analysis. The populations at these sites were both of mixed sex but varied in age with one containing fish around 18 months of age and the other containing fish around 24 months of age. Average water temperature and salinity from the sites on the day of sampling were 11.7 °C and 30.5 ppm, respectively. Individuals with an average weight of 3.5 kg were attracted through hand feeding and then taken out of the population using a scoop net and placed in a water bath containing a low dosage of tricaine mesylate (PHARMAQ, Hampshire, UK) to cause sedation. Blood was then sampled from the caudal vein using blood collection tubes (non‐vacuum sealed) and aliquoted into 1.3‐ml capacity tubes as specified in each investigation. Fish were then returned to their original pen.

Time from collection to separation of serum or plasma was standardized at 3 h (apart from investigation 2 where time to separation was the variable being studied). Separation was performed by centrifugation at 10 000 rcf for 3 min. For each investigation, paired samples were used with blood from the same fish being subjected to both treatment methods.

For each investigation, clinical biochemistry was performed by the Veterinary Diagnostic Service of the University of Glasgow, School of Veterinary Medicine. Concentrations or activities of albumin, alkaline phosphatase (ALP), alanine aminotransferase (ALT), amylase, aspartate aminotransferase (AST), calcium, chloride, cholesterol, CK, creatinine, gamma glutamyl transferase (GGT), globulin, glucose, LDH, lipase, magnesium, phosphate, potassium, sodium, total protein and albumin–globulin and sodium–potassium were assayed using Beckman Coulter assay kits (California, USA) following manufacturer instructions on an Olympus AU640 biochemical analyser (Beckmann Coulter, CA, USA).

### Investigation 1: effect of sample type

**Figure 1:**
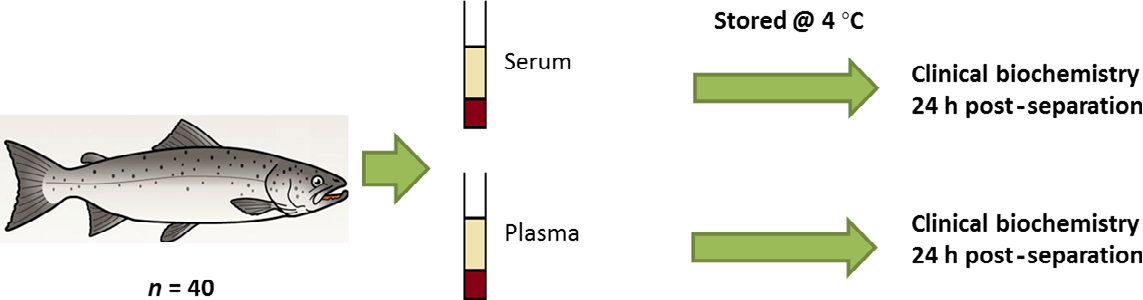
Schematic diagram of investigation 1 which investigated clinical biochemistry parameters in serum and plasma.

Blood from 40 salmon was split into plain collecting tubes and lithium heparin tubes for the collection of sera and plasma, respectively, from each individual fish. Samples were stored on wet ice during transport to the laboratory. On arrival, they were separated by centrifugation to serum or plasma 3 h post‐blood collection and stored at 4 °C, until clinical biochemistry was performed 24 h post‐separation of serum/plasma. This is represented as a schematic in Fig. 1.

### Investigation 2: effect of time to centrifugation and glucose stabilization

**Figure 2:**
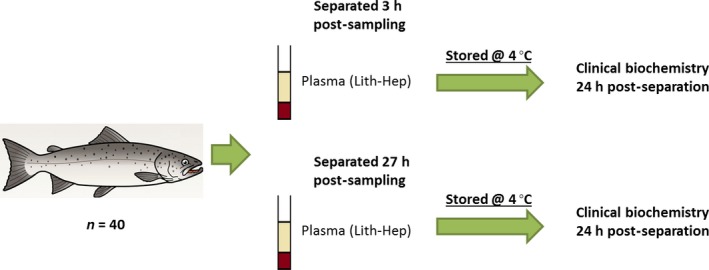
Schematic of investigation 2 which investigated the effect of time between blood collection and separation of plasma on biochemical parameters.

Individual blood samples collected from 40 fish were split into two lithium heparin blood tubes with one from each fish separated and plasma aspirated 3 h post‐collection and stored at 4 °C along with the unseparated tubes (see Fig. 2). Separated plasma was subjected to clinical biochemistry analysis 24 h post‐separation. The remaining tubes from each fish were centrifuged and plasma collected at 27 h post‐blood collection. These plasma samples were stored at 4 °C for a further 24 h and then analysed.

**Figure 3:**
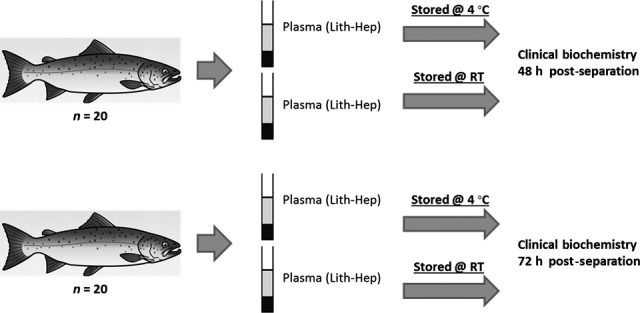
Schematic of investigation 3 where the effect of storage temperature for prolonged periods was studied.

**Figure 4:**
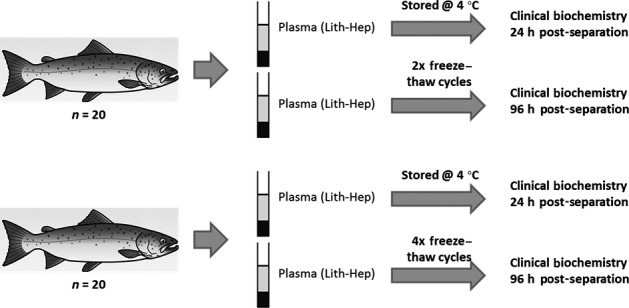
Schematic of investigation 4. Clinical biochemistries were analysed from samples after storage at 4 °C for 24 h or after 2x or 4x freeze–thaw cycles.

In addition, an aliquot of blood from all fish in this investigation was placed in fluoride oxalate tubes (recommended for glucose analysis) which were separated 3 h post‐blood collection, held at 4 °C, with glucose concentration determined 24 h post‐separation. These values were compared to glucose concentrations obtained from plasma from lithium heparin tubes which were treated in the same manner (Figs 3 & 4).

### Investigation 3 effect of post‐separation storage

Blood samples from 20 fish were aliquoted into two lithium heparin tubes which were centrifuged 3 h post‐blood collection. Plasma was then stored at either 4 °C or at room temperature (RT) for 48 h before biochemical analysis. Blood samples from a further 20 salmon were split and separated in the same manner but were held either at 4 °C or RT for 72 h prior to clinical biochemistry assays being performed.

### Investigation 4: effect of freeze–thaw cycles

Blood was collected from 20 fish and split into two lithium heparin tubes with plasma separated 3 h post‐collection. Half of the samples were stored at 4 °C and the other half subjected to two freeze–thaw cycles before clinical biochemistry was performed. Similarly, blood from twenty further fish was sampled and plasma split into two treatment groups that were either stored at 4 °C, or subjected to four freeze–thaw cycles.

### Statistical analyses

To analyse pre‐analytical treatment effects on clinical biochemistry parameters, results were compared between treatments in three ways to establish not only statistical significant differences but also in relation to clinical decision‐making. Paired results were analysed first by Wilcoxon signed‐rank test (WSRT) and then by paired *t*‐test and unpaired *t*‐test (GraphPad version 6, San Diego CA, USA).

### Reference intervals for biochemical analytes in salmon plasma

Reference ranges were established for the biochemical analytes of salmon plasma samples (*n* = 120) which had a normal (Gaussian) distribution by calculation of the values at plus and minus 2 standard deviations (SD) from the mean of the analyte values. When data was not normally distributed, which was the case for CK and LDH, the 2.5th and 97.5th percentiles were used as the reference range to include 95% of the sample results. The samples were collected and processed using the standardized protocol as described in the Results section of this study. The reference intervals were used to determine whether change in an analyte caused by the pre‐analytical treatments under investigation was sufficient to cause the analyte levels to be outside the reference interval limits and potentially lead to a false‐positive result for the analyte.

## Results

### Investigation 1: effect of tube type

Comparison of analytes in serum and plasma from the same salmon showed that the differences in the majority of clinical biochemistry analytes in plasma or serum were not significant. However, there were significant differences in phosphate, sodium and potassium concentrations between plasma and serum on the basis of the unpaired *t*‐tests (as well as paired *t*‐test and WSRT). Chloride and glucose levels were significant at the level of paired *t*‐test and WRST and albumin, ALP, calcium and total protein on the basis of WSRT exclusively. Table 1 shows the mean values of these analytes. In addition, results of statistical analysis are shown with p values from WSRT, paired and unpaired *t*‐tests. Salmon sera and plasma had undetectable activities of GGT and lipase.

**Table 1:** Mean analyte values from both plasma (*n* = 40) and sera (*n* = 40) in addition to *P* values from statistical analyses by Wilcoxon signed‐rank test (WSRT), paired *t*‐test and unpaired *t*‐test with significant results (*P* = <0.05) are indicated in **bold**; ‘*’ indicates that statistical analysis was not carried out due to insufficient paired sample numbers; ‘–’ indicates that statistical analysis was not carried out as the analyte was undetectable in all samples

Analyte	Plasma (±SE)	Sera (±SE)	WSRT	Paired *t*‐test	Unpaired *t*‐test
			*P* Value	*P* Value	*P* Value
Albumin (g/L)	14.08 (±0.31)	13.35 (±0.64)	**0.03846**	0.2952	0.3555
Albumin–Globulin (Ratio)	0.54 (±0.01)	0.52 (±0.02)	0.28014	0.5277	0.5151
ALP (U/L)	660 (±28.72)	665 (±32.3)	**0.03318**	0.5832	0.9281
ALT (U/L)	23.35 (±2.59)	23.25 (±2.96)	0.67448	0.8981	0.9833
Amylase (U/L)	957 (±62)	980 (±53.9)	0.08364	0.6487	0.7843
AST (U/L)	1435 (±194.62)	1399 (±243.31)	0.0784	0.9273	0.7469
Calcium (mmol/L)	3.15 (±0.029)	3.0 (±0.083)	**0.0009**	0.1059	0.1525
Chloride (mmol/L)	140.54 (+0.049)	142.3 (±0.91)	**0.02088**	**0.0124**	0.1004
Cholesterol (mmol/L)	6.97 (±0.18)	6.97 (±0.19)	0.5485	0.9861	0.9977
Creatine Kinase (U/L)	5022 (±1093.9)	5036 (±823.65)	*	*	*
Creatinine (umol/L)	22.57 (±0.0801)	21.96 (±0.96)	0.39532	0.3521	0.6834
GGT (U/L)	0	0	–	–	–
Globulin (g/L)	26.14 (±0.51)	26.18 (±0.74)	0.08544	0.9614	0.9686
Glucose (mmol/L)	4.51 (±0.14)	4.18 (±0.28)	**0.00148**	**0.0001**	0.1428
LDH (U/L)	1457 (±671)	1293 (198.32)	0.08914	0.4636	0.5364
Lipase (U/L)	0	0	–	–	–
Magnesium (mmol/L)	1.62 (±0.086)	1.29 (±0.18)	0.32218	0.1969	0.1251
Phosphate (mmol/L)	7.01 (±0.32)	8.47 (±0.39)	**0.00038**	**0.0001**	**0.0161**
Potassium (mmol/L)	0.81 (±0.06)	0.97 (±0.025)	**0.00278**	**0.0001**	**0.0002**
Sodium (mmol/L)	170 (±0.43)	173 (±0.79)	**0.00142**	**0.0012**	**0.0044**
Sodium–Potassium (Ratio)	215 (±5.64)	181 (±4.97)	**0.00386**	**0.0018**	**0.0002**
Total Protein (g/L)	40.67 (±0.67)	39.25 (±1.5)	**0.04444**	0.1054	0.2493

### Investigation 2: effect of time of separation and use of fluoride collecting tubes for glucose analysis

Extending the time of sample separation from 3 h to 27 h post‐blood collection caused a significant reduction in ALT, chloride and glucose by all tests of statistical significance used in the investigation. In contrast, calcium, magnesium, phosphate and potassium significantly increased in value. Smaller treatment effects were also observed on ALP, AST, cholesterol and CK with these only being found to be significantly different by WSRT analysis but not *t*‐tests due to population variability (Table 2). There was no significant difference in glucose concentration when using lithium heparin or fluoride blood collecting tubes (Table 3).

**Table 2:** Mean analyte values from plasma when separated from cells either 3 (*n* = 40) or 27 (*n* = 40) h post‐blood collection

Analyte	3 h (±SE)	27 h (±SE)	WSRT	Paired *t*‐test	Unpaired *t*‐test
			*P* value	*P* value	*P* value
Albumin (g/L)	16.17 (±0.16)	16.42 (±0.18)	0.13362	0.095	0.3253
Albumin–Globulin (Ratio)	0.508 (±0.004)	0.5055 (±0.004)	0.76418	0.7989	0.6564
ALP (U/L)	614 (±23.85)	633 (±23.68)	**0.00174**	0.4409	0.5737
ALT (U/L)	18 (±1.09)	11 (±0.62)	**0**	**0.0001**	**0.0001**
Amylase (U/L)	1059 (±49.06)	1113 (±49.32)	0.07346	0.9796	0.4537
AST (U/L)	760 (±76.62)	705 (±65.59)	**0.0027**	0.6653	0.5852
Calcium (mmol/L)	3.28 (±0.03)	3.40 (±0.03)	**0.00022**	**0.0001**	**0.0085**
Chloride (mmol/L)	133 (±0.47)	131 (±0.61)	**0.00262**	**0.0017**	**0.025**
Cholesterol (mmol/L)	10.70 (±0.24)	10.93 (±0.22)	**0.00174**	0.5163	0.5071
Creatine Kinase (U/L)	9025 (±968.46)	10803 (±1018.8)	**0.02642**	0.2619	0.2183
Creatinine (umol/L)	32 (±1.76)	30 (±1.58)	0.17068	0.6823	0.5175
GGT (U/L)	0	0	–	–	–
Globulin (g/L)	31 (±0.37)	32 (±0.39)	**0.00906**	**0.0062**	0.198
Glucose (mmol/L)	5.51 (±0.12)	2.94 (±0.14)	**0**	**0.0001**	**0.0001**
LDH (U/L)	2233 (±396.54)	1990 (±300.55)	0.09894	0.2385	0.6207
Lipase (U/L)	0	0	–	–	–
Magnesium (mmol/L)	1.53 (±0.06)	2.19 (±0.06)	**0**	**0.0001**	**0.0001**
Phosphate (mmol/L)	5.8 (±0.26)	7.9 (±0.41)	**0.0003**	**0.0003**	**0.0001**
Potassium (mmol/L)	0.889 (±0.02)	0.983 (±0.03)	**0.02202**	**0.0272**	**0.0163**
Sodium (mmol/L)	166 (±0.64)	167 (±0.61)	0.93624	0.8905	0.8149
Sodium–Potassium (Ratio)	189 (±3.18)	175 (±5.2)	**0.0455**	0.0649	**0.0445**
Total Protein (g/L)	48 (±0.49)	49 (±0.55)	**0.00528**	**0.0072**	0.2059

Results of Wilcoxon signed‐rank test (WSRT), paired *t*‐test and unpaired *t*‐test statistical analyses with significant differences (*P* = <0.05) indicated in **bold**; ‘–’ indicates that statistical analysis was not carried out as the analyte was undetectable in all samples.

**Table 3:** Mean glucose concentrations when using lithium heparin (lith = hep) (*n* = 40) or fluoride‐coated blood collecting tubes (*n* = 40) and *P* values of Wilcoxon signed‐rank test (WSRT), and paired and unpaired *t*‐tests

Analyte	Tube Type	Fluoride (±SE)	WSRT	Paired *t*‐test	Unpaired *t*‐test
	Lithium Heparin (±SE)		*P* Value	*P* Value	*P* Value
Glucose (mmol/l)	5.51 (±0.12)	5.72 (±0.14)	0.11876	0.094	0.248

### Investigation 3: effect of storage temperature and time

Storage of samples for 48 (Table 4) h at room temperature significantly reduced the activities of ALP, ALT, AST and CK, and the concentration of calcium, whereas concentrations of glucose, creatinine, magnesium, phosphate and total protein increased significantly by both paired *t*‐test and WSRT. These differences were generally small but increased further after 72 h at RT (Table 5) with ALP, ALT, AST and CK activities and calcium concentration all being at lower activity/concentration in samples stored at room temperature and with sodium lower only in the WSRT statistical test. Only creatinine and phosphate were at significantly higher concentrations in samples stored at room temperature for 72 h.

**Table 4:** Effect of storage of plasma at 4 °C (*n* = 20) or room temperature (RT) (*n* = 20) for 48 h

48 h	4 °C (±SE)	RT (±SE)	WSRT	Paired *t*‐test	Unpaired *t*‐test
Analyte			*P* Value	*P* Value	*P* Value
Albumin (g/L)	15.35 (±0.264)	15.6 (±0.266)	*	0.0563	0.5087
Albumin–Globulin (Ratio)	0.495 (±0.007)	0.497 (±0.005)	*	0.6557	0.7775
ALP (U/L)	629 (±33.3)	621 (±32.99)	**0.00854**	**0.0095**	0.8529
ALT (U/L)	9.4 (±0.822)	6 (±0.497)	**0.00014**	**0.0001**	**0.0011**
Amylase (U/L)	1033 (±61)	1038 (±61.8)	0.17068	0.1114	0.9553
AST (U/L)	724.6 (±107.8)	501.1 (±68.9)	**0.00008**	**0.0001**	0.0876
Calcium (mmol/L)	3.32 (±0.03)	3.28 (±0.034)	**0.00132**	**0.0006**	0.339
Chloride (mmol/L)	133.19 (±0.49)	132.3 (±0.509)	0.07672	0.0533	0.2159
Cholesterol (mmol/L)	10.91 (±0.289)	10.93 (±0.292)	0.4413	0.5612	0.9586
Creatine Kinase (U/L)	9183 (±1530)	7704 (±1277)	**0.00008**	**0.0001**	0.4627
Creatinine (umol/L)	29.4 (±1.98)	31.45 (±2.06)	**0.00062**	**0.0001**	0.4777
GGT (U/L)	0	0	–	–	–
Globulin (g/L)	31.1 (±0.542)	31.4 (±0.596)	0.09296	0.0553	0.7117
Glucose (mmol/L)	5.15 (±0.155)	5.23 (±0.156)	**0.02382**	**0.0095**	0.7176
LDH (U/L)	1116 (±214)	1077 (±188.7)	0.82588	0.4782	0.892
Lipase (U/L)	0	0	–	–	–
Magnesium (mmol/L)	1.82 (±0.027)	1.87 (±0.026)	**0.0012**	**0.0005**	0.2108
Phosphate (mmol/L)	7.34 (±0.168)	8.59 (±0.257)	**0.00008**	**0.0001**	**0.0003**
Potassium (mmol/L)	0.82 (±0.016)	0.855 (±0.017)	0.11642	0.0593	0.1368
Sodium (mmol/L)	165 (±0.356)	166 (±0.498)	0.08012	0.0578	0.0945
Sodium–Potassium (Ratio)	203 (±4.053)	196 (±4.143)	0.24604	0.1266	0.2376
Total Protein (g/L)	46.45 (±0.745)	47 (±0.83)	**0.00512**	**0.0007**	0.6248

Results of Wilcoxon signed‐rank test (WSRT), paired *t*‐test and unpaired *t*‐test statistical analyses with significant differences (*P* = <0.05) indicated in **bold**; ‘*’ indicates that statistical analysis was not carried out due to insufficient paired sample numbers; ‘–’ indicates that statistical analysis was not carried out as the analyte was undetectable in all samples.

**Table 5:** Effect of storage of plasma at 4 °C (*n* = 20) or room temperature (RT) (b = 20) for 72 h

72 h	4 °C (±SE)	72 h (±SE)	WSRT	Paired *t*‐test	Unpaired *t*‐test
Analyte			*P* Value	*P* Value	*P* Value
Albumin (g/L)	14.9 (±0.26)	15.2 (±0.312)	*	0.0828	0.4658
Albumin–Globulin (Ratio)	0.48 (±0.006)	0.5 (±0.009)	0.14706	0.1345	0.2048
ALP (U/L)	491 (±34.4)	482 (±33.5)	**0.00932**	**0.0072**	0.8556
ALT (U/L)	8.45 (±1.29)	4.4 (±0.694)	**0.00028**	**0.0001**	**0.0087**
Amylase (U/L)	847 (±58.2)	849 (±58.217)	0.57548	0.5113	0.9831
AST (U/L)	854 (±171)	487 (±94)	**0.00008**	**0.0002**	0.0688
Calcium (mmol/L)	3.19 (±0.034)	3.15 (±0.035)	**0.01108**	**0.0174**	0.3791
Chloride (mmol/L)	131 (±0.537)	130 (±0.471)	0.22628	0.2369	0.5462
Cholesterol (mmol/L)	9.9 (±0.333)	9.8 (±0.331)	0.08544	0.0692	0.839
Creatine Kinase (U/L)	4709 (±385)	3840 (±1270)	**0**	**0.0002**	0.0907
Creatinine (umol/L)	32.9 (±3.27)	36.4 (±3.601)	**0.0002**	**0.0087**	0.4763
GGT (U/L)	0	0	–	–	–
Globulin (g/L)	30.65 (±0.563)	30.4 (±0.564)	0.32708	0.2617	0.7555
Glucose (mmol/L)	5.16 (±0.106)	4.9 (±0.241)	0.5157	0.3928	0.4091
LDH (U/L)	1278 (±298)	1410 (±370)	0.05238	0.1275	0.782
Lipase (U/L)	0	0	–	–	–
Magnesium (mmol/L)	1.59 (±0.0231)	1.61 (±0.0232)	0.13622	0.1863	0.6075
Phosphate (mmol/L)	6.31 (±0.136)	7.34 (±0.259)	**0.0001**	**0.0001**	**0.0012**
Potassium (mmol/L)	0.79 (±0.0203)	0.81 (±0.0216)	0.57548	0.3299	0.5053
Sodium (mmol/L)	163 (±0.524)	164 (±0.411)	**0.03846**	0.0808	0.3579
Sodium–Potassium (Ratio)	209 (±5.007)	205 (±5.2601)	0.92828	0.4325	0.5729
Total Protein (g/L)	45 (±0.786)	45 (±0.789)	*	0.7481	0.9644

Results of Wilcoxon signed‐rank test (WSRT), paired *t*‐test and unpaired *t*‐test statistical analyses with significant differences (*P* = <0.05) indicated in **bold**; ‘*’ indicates that statistical analysis was not carried out due to insufficient paired sample numbers; ‘–’ indicates that statistical analysis was not carried out as the analyte was undetectable in all samples.

### Investigation 4: effect of freeze–thaw cycles

Freezing and thawing samples was found to have significant effects on albumin, globulin, ALP, amylase, AST, calcium, chloride, cholesterol, creatinine, phosphate, potassium, sodium and total protein. In most cases, differences between control and those freeze–thawed increased between 2x (Table 6) and 4 × cycles (Table 7). Moreover, while 2x freeze–thaw cycles did not cause a significant change in ALT by 4x freeze–thaw cycles, its activity dropped significantly. In addition, most differences between control and 2x freeze–thaw cycles, while being significantly different by WSRT and paired *t*‐test, were not different by the unpaired *t*‐test. This indicated that any effect of a 2x freeze–thaw cycle would not significantly alter interpretation of results from two independent studies or be clinically significant. However, after 4x cycles of freeze–thaw, more analytes were significantly different by unpaired *t*‐tests due to increasing differential when compared to control samples.

**Table 6:** Mean analyte concentrations from samples treated to control conditions (*n* = 20) and those subjected to two freeze–thaw cycles (2x) (*n* = 20)

Analyte	Control	2x	WSRT	Paired *t*‐test	Unpaired *t*‐test
			*P* Value	*P* Value	*P* Value
Albumin (g/L)	15.4 (±0.266)	16.15 (±0.319)	**0.00096**	**0.0001**	0.0784
Albumin–Globulin (Ratio)	0.527 (±0.007)	0.484 (±0.006)	**0.0003**	**0.0001**	**0.0001**
ALP (U/L)	590 (±24.09)	628 (±26. 06)	**0.00012**	**0.0001**	0.288
ALT (U/L)	15.3 (±1.29)	15.65 (±1.34)	0.28014	0.2601	0.8522
Amylase (U/L)	1006 (±56.4)	1070 (±60.4)	**0.00008**	**0.0001**	0.4432
AST (U/L)	686 (±50.9)	724 (±57.1)	**0**	**0.0011**	0.6147
Calcium (mmol/L)	3.31 (±0.02)	3.37 (± 0.03)	**0.03846**	**0.0253**	0.1095
Chloride (mmol/L)	131 (±0.52)	133 (±0.911)	**0.00694**	**0.0045**	**0.0181**
Cholesterol (mmol/L)	11.08 (±0.333)	11.43 (±0.353)	**0.00152**	**0.001**	0.4812
Creatine Kinase (U/L)	8713 (±1213)	10437 (±1517)	**0.00008**	**0.0001**	0.3805
Creatinine (umol/L)	29.9 (±2.65)	31.95 (±2.58)	**0.00164**	**0.0018**	0.5822
GGT (U/L)	0	0	–	–	–
Globulin (g/L)	29.35 (±0.549)	33.4 (±0.689)	**0.00008**	**0.0001**	**0.0001**
Glucose (mmol/L)	5.465 (±0.178)	5.51 (±0.169)	0.29372	0.2428	0.856
LDH (U/L)	1021 (±132)	1058 (±134)	0.11642	0.3134	0.8465
Lipase (U/L)	0	0	–	–	–
Magnesium (mmol/L)	1.71 (±0.0368)	1.747 (±0.0334)	**0.01046**	**0.0106**	0.4938
Phosphate (mmol/L)	6.299 (±0.107)	6.568 (±0.115)	**0.0009**	**0.0002**	0.0944
Potassium (mmol/L)	0.815 (±0.182)	0.92 (±0.186)	**0.00096**	**0.0001**	**0.0003**
Sodium (mmol/L)	163.19 (±0.422)	165.75 (±0.907)	**0.01878**	**0.0168**	**0.0146**
Sodium–Potassium (Ratio)	202 (±4.645)	181 (±3.28)	**0.001**	**0.0003**	**0.0008**
Total Protein (g/L)	44 (±0.489)	49 (±0.966)	**0.0002**	**0.0001**	**0.0001**

Results of Wilcoxon signed‐rank test (WSRT), paired *t*‐test and unpaired *t*‐test statistical analyses with significant differences (*P* = <0.05) indicated in **bold**; ‘–’ indicates that statistical analysis was not carried out as the analyte was undetectable in all samples.

**Table 7:** Mean analyte concentrations from samples treated to control conditions (*n* = 20) and those subjected to four freeze–thaw cycles (4x) (*n* = 20)

Analyte	Control (±SE)	4x (±SE)	WSRT	Paired *t*‐test	Unpaired *t*‐test
			*P* Value	*P* Value	*P* Value
Albumin (g/L)	15.45 (±0.235)	16.45 (±0.246)	**0.00044**	**0.0001**	**0.0001**
Albumin–Globulin (Ratio)	0.53 (±0.006)	0.49 (±0.006)	**0.00014**	**0.0001**	**0.0001**
ALP (U/L)	592 (±19.47)	639.95 (±24.25)	**0.00168**	**0.0015**	0.1314
ALT (U/L)	13.8 (±1.033)	11.9 (±0.849)	**0.0003**	**0.0001**	0.1746
Amylase (U/L)	1069 (±49.7)	1148 (±48.1)	**0.001**	**0.0011**	0.2443
AST (U/L)	654 (±64.74)	699 (±70)	**0.00452**	**0.0019**	0.6393
Calcium (mmol/L)	3.3 (±0.024)	3.44 (±0.063)	0.08544	**0.0478**	0.0562
Chloride (mmol/L)	131 (±0.339)	136 (±2.2)	**0.05**	**0.0319**	**0.038**
Cholesterol (mmol/L)	11.03 (±0.248)	11.57 (±0.334)	**0.01878**	**0.0171**	0.2029
Creatine Kinase (U/L)	9114 (±1874.1)	11199 (±2269)	**0.0002**	0.4184	0.4264
Creatinine (umol/L)	28.4 (±2.389)	31.1 (±2.518)	**0.01078**	**0.0097**	0.4499
GGT (U/L)	0	0	–	–	–
Globulin (g/L)	29.05 (±0.53)	33.75 (±0.817)	**0.00014**	**0.0001**	**0.0001**
Glucose (mmol/L)	5.07 (±0.137)	5.18 (±0.165)	0.3125	0.2481	0.6114
LDH (U/L)	1396 (±273.05)	1557 (±325.8)	0.2113	0.1792	0.7076
Lipase (U/L)	0	0	–	–	–
Magnesium (mmol/L)	1.69 (±0.016)	1.77 (±0.04)	0.07346	0.0532	0.0856
Phosphate (mmol/L)	6.33 (±0.342)	7.2 (±0.253)	**0.0048**	**0.0303**	**0.049**
Potassium (mmol/L)	0.81 (±0.0191)	0.91 (±0.017)	**0.00222**	**0.0002**	**0.0006**
Sodium (mmol/L)	163 (±0.427)	169 (±2.897)	**0.03846**	**0.0272**	**0.0351**
Sodium–Potassium (Ratio)	203 (±4.848)	188 (±4.616)	**0.0151**	**0.0086**	**0.0304**
Total Protein (g/L)	45 (±0.4651)	50 (±1.043)	**0.00044**	**0.0004**	**0.0003**

Results of Wilcoxon signed‐rank test (WSRT), paired *t*‐test and unpaired *t*‐test statistical analyses with significant differences (*P* = <0.05) indicated in **bold**; ‘–’ indicates that statistical analysis was not carried out as the analyte was undetectable in all samples.

### Reference intervals for clinical biochemistry analytes

Reference intervals (Table 8) for 22 biochemical analytes in salmon plasma were established using 120 samples which had been collected by the optimized protocol outlined below.

**Table 8:** Reference ranges of analytes from samples treated using the standardized protocol (*n* = 120)

Analyte	Reference Range
Albumin (g/L)	13–17
Albumin–Globulin (Ratio)	0.46–0.58
ALP (U/L)	380–821
ALT (U/L)	4.54–27.89
Amylase (U/L)	560–1534
AST (U/L)	45–1371
Calcium (mmol/L)	3–3.45
Chloride (mmol/L)	128–136
Cholesterol (mmol/L)	8.3–13.5
Creatine Kinase (U/L)	**2258–20567**
Creatinine (umol/L)	9.3–51
GGT (U/L)	<0.01
Globulin (g/L)	25–35
Glucose (mmol/L)	4–6.2
LDH (U/L)	**302–5579**
Lipase (U/L)	<0.01
Magnesium (mmol/L)	1.2–2
Phosphate (mmol/L)	3.5–8.5
Potassium (mmol/L)	0.665–1.023
Sodium (mmol/L)	158–170
Sodium–Potassium (Ratio)	156–238
Total Protein (g/L)	40–52

The reference ranges in **bold** were calculated by the 2.5–97.5 percentile method; otherwise, they were calculated on the basis of the mean ± 2 SD.

### Protocol for sample collection and pre‐analytical handling

From the results of this investigation, a sampling and processing protocol for salmon blood samples prior to biochemical analysis is proposed:

1. Collect blood from the caudal vein using a non‐vacuum‐sealed blood collection tube and immediately transfer into 1.3‐mL capacity lithium heparin tube.
2. Centrifuge at 10 000 g for 3 min to separate plasma from the blood cells and transfer this plasma to a labelled storage tube and put on wet ice at 4 °C during transfer.
3. Analyse plasma biochemistry at 24 h post‐sampling.
4. In laboratory store at 4 °C before analysis.
5. After analysis, samples can be stored at −20 °C or −80 °C for any further work or retesting.

## Discussion

This study has shown that pre‐analytical treatment of salmon plasma or serum can cause significant variability in clinical biochemistry analytes. The three types of statistical analysis used in this study were employed to test significance at varying levels of sensitivity allowing assessment of treatment effects on biochemical results. Wilcoxon signed‐rank test which is the most sensitive of these could identify small but significant treatment effects. Significance solely from this test indicates that while treatment may be having an effect on the given analyte, it is small and may not be significant when all paired results are compared, that is the population of results. This significance is given by paired *t*‐test analysis, with such results indicating that the treatment effects may alter an analytes concentration significantly at a population level. In the analytes where standard error of the mean was high, tests for such analytes required larger changes compared to WSRT to indicate significance this was evident, for example, in CK which showed high individual variation in all investigations. To establish whether any treatment effects cause a different clinical decision or introduce significant change between differently treated samples, the results were also treated as unrelated to simulate the comparison of two independent population results, and tested by unpaired *t*‐tests.

Investigations were designed to provide information on sample type effects (serum v plasma) and also on various pre‐analytical treatment effects on the specimens. There was little difference in clinical biochemistry results between serum and plasma. It is apparent that the optimal specimen and tube type is still debated in human and veterinary medicine (Humann‐Ziehank & Ganter 2012). However, serum was found to have, at all levels of significance, higher phosphate, potassium and sodium activities. This indicates that sample sets, as part of health monitoring, must exclusively use one fluid type when determining electrolyte concentrations. In the subsequent investigations, plasma was the sample of choice so as not to exclude fibrinogen and other coagulation cascade proteins and also so that there would be no need to allow time for coagulation for serum samples.

Sample treatment was found to have a much greater effect on biochemistry results than the type of specimen. For instance, the time elapsed from blood collection to separation of plasma via centrifugation was found to alter certain analyte activities. Alanine transaminase, calcium, chloride, globulin, glucose, magnesium, phosphate, potassium and total protein altered significantly when 72 h had elapsed before separation. Indeed, Humann‐Ziehank & Ganter (2012) advised that in farm animals, separation of blood from farm animals should be performed within 1 h of sampling. The reasons for this included: release of CK from cells, leakage of potassium and phosphate from erythrocytes and effects of glycolysis. However, a sampling protocol involving a 1‐h window between time of sample collection and separation of plasma from blood cells may be difficult to follow in aquaculture. Significant differences in analyte levels between samples separated 3 h and 27 h post‐sampling are less evident, but time before separation should be minimized as in the standardized protocol suggested in this study.

Salmon plasma, collected with the proposed standardized protocol, yielded similar ion concentrations as reported by Bepgheim *et al*. (1990). Although in their study of salmon in Norway, blood was sampled by heart puncture, it indicates that neither the anatomical nor geographic site of blood sampling significantly affects the ion concentrations. Mean concentrations or enzyme activities in salmon serum reported by Sandnes, Lie & Waagbo (1988) were within the reference ranges established here (Table 8) for all analytes apart from ALP, albumin and albumin–globulin ratio. In the previous study, the mean ALP was 853 IU/L which was close to the upper limit of the reference range (380–821 IU/L), while the mean albumin was 20 g/L, which is not far outside the reference interval found here (13–17 g/L). The albumin–globulin ratio was 0.41 in the previous study but 0.46–0.58 in the present study. There could be technical reasons for these differences. For assay of enzymes such as ALP, variation in results between laboratories can be caused by different methodologies provided by the diagnostic kit manufacturers or by the temperature of the assays. Albumin assays could vary depending on the use of bromocresol purple or bromocresol green as dye binding reagent. Considering the 27 years between the studies, geographic differences in sampling sites, season and sea temperature of sampling days and assay variations, there is substantial agreement between the current and previous studies. It is considered good practice for a diagnostic laboratory to establish reference ranges based on their own methods and instrumentation. Future work on the use of the reference ranges is required to assess the biological variation caused by smolt type, life stage, genetics, feeds and site effects.

Storage temperature of samples is an important consideration for transit of samples and after receiving them in laboratories. However, there is no universal recommendation of temperature and duration of storage, although it has been shown that many biochemical analytes are relatively stable after 24–48 h of storage at RT (Braun *et al*. 2015). Our present study found that prolonged storage at RT compared with storage at 4 °C did affect a number of analytes including ALP, ALT, AST, calcium, CK, creatinine and phosphate. While this treatment caused effects by 48 h, the effect of storage at RT became more pronounced by 72 h. However, only ALT and phosphate levels were altered sufficiently to be significant by unpaired *t*‐test. Thus, while storing samples at room temperature has an effect on some analytes, many remain stable for as long as 72 h. Nevertheless, it is recommended that storage at 4 °C is preferable where possible.

Storage temperature in veterinary clinical biochemistry can vary, though commonly, samples for repeat analysis or long‐term storage are frozen (Reynolds *et al*. 2006). Previous studies have found that the impacts of freezing are variable depending on specific analytes in question. The number of freeze–thaw cycles and the time elapsed between freeze–thaw cycles have differential effects on parameters (Thoresen *et al*. 1995; Braun *et al*. 2015). Through subjecting salmon plasma samples to 4 °C and either 2x or 4x freeze–thaw cycles, it was found that most analytes were sensitive to freeze–thawing although often statistical significance was restricted to WRST while paired *t*‐test analysis indicated that such results would not be clinically significant. Similar results have been found by Reynolds *et al*. in 2006 who found no clinically significant changes in biochemistries after 3x freeze–thaw cycles of canine plasma. Despite this, differences between paired salmon plasma samples either stored at 4 °C or subject to freezing were more pronounced after 4x freeze–thaw cycles for albumin, globulin, phosphate, potassium, sodium and total protein levels. These were all significantly different, with all statistical tests used. Cyclical freezing is widely accepted to denature proteins (Humann‐Ziehank & Ganter 2012) which would presumably reduce clinical biochemical values due to a reduction in activity; in this study, total protein increased after freeze–thawing and this observation has been reported in bovine plasma (Proverbio *et al*. 2015) and equine serum (Alberghina *et al*. 2013). While these previous studies gave no explanation as to why this may have been observed, it is not likely that the freeze–thaw cycles can increase the amount of protein in plasma. It could be possible that freezing affects the proteins in a way to increase the biuret reaction, used to measure total protein. Perhaps a set of peptide bonds not previously available for the biuret reaction are exposed after thawing. Alternatively, evaporation during this process could cause concentration of samples.

Establishing reference intervals for analysis in this study is valuable as little work has been carried out previously in Atlantic salmon. However, while this investigation is more extensive than previous studies, the ranges given must be used with caution. While samples used are from two different sites and were sampled when no diseases were present, a clinically healthy reference interval must also take into account as much biological variation as possible. These were useful in investigating effects at a clinical level, demonstrating that while many technical pre‐analytical treatments lead to statistically significant differences, they are often not large enough to be clinically significant being still within the reference range for the particular analyte. Nevertheless, delay of plasma and cell separation was found to alter magnesium and glucose levels, while storage at room temperature reduced ALT concentrations out with reference range making them clinically relevant findings.

This study has highlighted that clinical biochemistry parameters are sensitive to a number of different treatments, which must be considered in the future if blood sampling is to increase in use during disease screening in salmon. However, the significance of these pre‐analytical treatments is variable depending on the statistics or criteria used. While there have been few previous investigations of clinical biochemical changes during disease in salmon, it can be a pivotal aid for the aquaculture industry in adapting a more proactive and welfare conscious approach to health monitoring. Previous studies have highlighted the potential use of individual biochemical analysis. For example, lipase activity has been shown to be a marker of pathological damage caused by pancreas disease (Grant, Brown & Laider 1994; McCoy *et al*. 1994). However, McCoy *et al*. (1994) showed that basal plasma concentrations were negligible in most individuals which would explain the lack of lipase activity in any samples from this study. It is proposed that the case here is similar for GGT where the activity was too low for the sensitivity of the test in circulating plasma of healthy salmon. Investigations in diseased salmon may show, as for lipase, that this increases during pathological damage. Therefore, future work must assess the biomarker potential of many of these clinical biochemistry parameters in Atlantic salmon, which are well established in other areas of veterinary and human medicine. This study has shown that pre‐analytical treatments vary in their effects on biochemical analytes. Furthermore, it has also shown that establishing a standardized system for sample collection and processing would be advantageous for widespread aquaculture health monitoring. Such a system would prevent false diagnosis of health status and also limit analytical variability between and within populations.

## Conclusion

Clinical biochemistry is an established and valuable methodology for health monitoring in livestock and domestic animals. However, such an approach has not been widespread in aquaculture. Therefore, this study has aimed to propose a standardized sampling protocol while investigating the effects of pre‐analytical treatment of specimens to inform best practice in the future. It has highlighted how differential treatment can lead to variation in a number of analytes, thus making it essential for standardized monitoring to be adopted. Future work must aim to establish the effects of biological pre‐analytical causes for variation, such as diet, stress and site effects. Moreover, it has highlighted the profound effect that post‐analytical interpretation has on what we see as being ‘significant’ treatment effects. In addition, investigations in the changes in biochemical analytes during diseases and other treatments are required to truly establish the diagnostic potential of this approach. This would complement initiatives to discover specific biomarkers, as well as to develop tools for assessing efficacy of treatments, vaccines and protective/functional feeds which are widespread within the industry.
